# Candidiasis by *Candida glabrata*, *Candida nivariensis* and *Candida bracarensis* in *Galleria mellonella*: Virulence and Therapeutic Responses to Echinocandins

**DOI:** 10.3390/jof7120998

**Published:** 2021-11-23

**Authors:** Ainara Hernando-Ortiz, Elena Eraso, Guillermo Quindós, Estibaliz Mateo

**Affiliations:** Department of Immunology, Microbiology and Parasitology, Faculty of Medicine and Nursing, University of the Basque Country (UPV/EHU), P.O. Box 699, 48080 Bilbao, Spain; ainara.hernando@ehu.eus (A.H.-O.); elena.eraso@ehu.eus (E.E.); guillermo.quindos@ehu.eus (G.Q.)

**Keywords:** emerging pathogen, pathogenesis, antifungal susceptibility, invertebrate models

## Abstract

*Candida albicans* is the major etiological agent of invasive candidiasis but the increasing prevalence of emerging species of *Candida*, such as *Candida glabrata* and phylogenetically closely related species, *Candida nivariensis* and *Candida bracarensis*, requires special attention. Differences in virulence among these species and their therapeutic responses using in vivo non-mammalian models are scarcely analysed. The aim of this study was analyse the survival of *G. mellonella* and host-pathogen interactions during infection by *C. glabrata*, *C. nivariensis* and *C. bracarensis*. Moreover, therapeutic responses to echinocandins were also assessed in the *G. mellonella* model of candidiasis. These three species produced lethal infection in *G. mellonella*; *C. glabrata* was the most virulent species and *C. bracarensis* the less. Haemocytes of *G. mellonella* phagocytised *C. bracarensis* cells more effectively than those of the other two species. Treatment with caspofungin and micafungin was most effective to protect larvae during *C. glabrata* and *C. nivariensis* infections while anidulafungin was during *C. bracarensis* infection. The model of candidiasis in *G. mellonella* is simple and appropriate to assess the virulence and therapeutic response of these emerging *Candida* species. Moreover, it successfully allows for detecting differences in the immune system of the host depending on the virulence of pathogens.

## 1. Introduction

There is a substantial change in the aetiology of candidiasis worldwide, with an increasing prevalence of non-*Candida albicans* species, such as *Candida parapsilosis*, *Candida glabrata*, *Candida tropicalis*, *Candida krusei* and *Candida auris*. *C. glabrata* is an emerging pathogen and the second cause of candidaemia in the USA, Canada, Australia and Northern and Eastern Europe. In Latin America, Africa, and the European Mediterranean countries such as Spain, candidiasis due to *C. glabrata* are the third most frequent, behind those caused by *C. albicans* and *C. parapsilosis* [1]. *Candida bracarensis* and *Candida nivariensis* are species phylogenetically close to *C. glabrata* that should be identified by molecular methods, including MALDI-TOF MS (Matrix Assisted Laser Desorption/Ionization Mass Spectrometry), due to their high phenotypic similarities and genetic closeness [2,3,4,5].

*C. glabrata* often develops resistance to widely used azoles, especially fluconazole [6]. Since the echinocandins, anidulafungin (AND), caspofungin (CAS), and micafungin (MCF), present high activity against *C. glabrata*, there are the treatment of choice for *C. glabrata* invasive infections. These antifungal drugs inhibit the synthesis of cell wall β-1,3-glucan, which is encoding in *FKS1*, *FKS2*, and *FKS3* genes. However, echinocandin resistance, associated with *FKS* genes mutations, has been reported in *C. glabrata* [7,8,9,10], specifically in the hot spot mutations of *FKS1* and *FKS2* genes [6,11,12]. Moreover, differences in the expression of these two *FKS* genes were detected between young and old *C. glabrata* cells [13]. Besides point mutations, overexpression of *FKS* genes were also noted in 14-generation-old cells of *C. glabrata* resistant to MCF and in the biofilms formed by this specie after contact with MCF [13,14]. Frequency of *C. nivariensis* and *C. bracarensis* candidiasis is low (0.05–0.2%) and evidence on echinocandins activities against these species is scarce [15,16,17]. However, it is necessary a deeper knowledge to avoid therapeutic failures [18].

Common mammalian models of infection are linked to ethical concerns that recommend limiting their use. Models in invertebrate animals, such as those developed in *Caenorhabditis elegans* (*Rhabditida*: *Rhabditidae*) and in *Galleria mellonella* (*Lepidoptera: Pyralidae*), are being increasingly introduced for studying host-pathogen interactions and for evaluating antimicrobial efficacy of conventional antimicrobial agents and new molecules [18,19,20,21]. The caterpillar larvae of *G. mellonella*, commonly known as the greater wax moth, is a useful model for studying host-pathogen interactions because its immune system has conserved similarities with the mammal innate defences [22,23,24]. These interactions can be evaluated by assessing the larvae response against pathogens and the phagocytic capacity of haemocytes present in the haemolymph of the larvae [25,26]. Furthermore, the *G. mellonella* model of candidiasis has been successfully applied to monitor the difference virulence between young and old cells of *C. glabrata* [27,28].

In this work, we analyse the virulence of *C. glabrata*, *C. nivariensis* and *C. bracarensis* in *G. mellonella*, and the interactions between these *Candida* species and the *G. mellonella* haemocyte density and phagocytic response. Moreover, the effectiveness of echinocandins, AND, CAS, and MCF, for treating invasive candidiasis caused by these *Candida* species was evaluated in the *G. mellonella* host model.

## 2. Materials and Methods

### 2.1. Candida Strains and Growth Conditions

Six commercially available reference strains, including two strains of each species *C. glabrata*, *C. nivariensis* and *C. bracarensis*, were obtained from different culture collections (Table 1).

Yeasts were cultured overnight in yeast extract peptone dextrose broth (YEPD; 1% yeast extract, 2% bacteriological peptone, 2% d-glucose) medium (Panreac, Spain) at 30 °C under shaking conditions. Then, yeast cells were washed three times with phosphate-buffered saline solution (PBS) and resuspended in PBS supplemented with ampicillin (20 mg/L) to prevent infection with bacteria naturally present on the surface of *G. mellonella* larvae. Cell counting was performed by microscopy using a Burker haemocytometer and three concentrations of 1 × 10^7^, 1 × 10^8^ and 1 × 10^9^ yeast cells/mL were prepared in PBS-ampicillin (20 mg/L) to use as inocula.

### 2.2. Survival of Galleria mellonella

Larvae of *G. mellonella* weighing between 0.3 and 0.5 g (Bichosa, Spain) were placed in groups of 20 individuals in Petri plates to perform the experiments. The last left pro-leg of larvae was cleaned with ethanol 70% before injecting 10 μL of *Candida* suspension into the larvae haemocele with a precision syringe (ref. P/N 5190-1493, Agilent, Santa Clara, CA, USA). The final inocula tested were 1 × 10^5^, 1 × 10^6^ and 1 × 10^7^ cells/larva. Two uninfected larvae groups were used as controls in all trials: a group of untouched larvae and a group of larvae injected with 10 µL of PBS-ampicillin to control the possible impact of the injection and the effect of the PBS-ampicillin buffer on larvae survival (sham group). The larvae were incubated at 37 °C in dark for 120 h, and survival was monitored every 24 h by visual inspection of melanisation and the absence of movement. Each trial was performed at least three times on different days. A total of 180 larvae were used to assess the infection caused for each of the six *Candida* strains and 40 larvae were used as control in each trial.

### 2.3. Haemocyte Density Determination

Groups of five larvae of *G. mellonella* were inoculated with 1 × 10^5^, 1 × 10^6^ and 1 × 10^7^ cells/larva. As control, a group of uninfected larvae inoculated with 10 µL PBS-ampicillin was used (sham group). Larvae were incubated at 37 °C in dark for 3 h and then, 50 µL of haemolymph was collected from each larva and mixed with insect physiological saline buffer (IPS buffer; 150 mM sodium chloride, 5 mM potassium chloride, 10 mM Tris-HCl pH 6.9, 10 mM EDTA and 30 mM sodium citrate) to avoid melanisation and coagulation of haemolymph. Haemocyte density was determined by microscopy counting using a haemocytometer. Each assay was performed at least three times on different days.

### 2.4. Phagocytic Activity of Haemocytes

Five *G. mellonella* larvae were used for each *Candida* strain. Yeast cells were stained with 0.4 mg/mL of Calcofluor white (Sigma Aldrich, St. Louis, MO, USA) for 30 min at 30 °C and washed twice with PBS before inoculated in each larva 1 × 10^6^ cells/larva. Larvae were incubated at 37 °C for 2 h and afterwards, 50 µL of haemolymph of each larva was collected in the same volume of IPS buffer. Phagocytosis quantification was performed by fluorescence microscope Nikon Eclipse 80i (Nikon, Tokyo, Japan) counting a minimum of 100 haemocytes with and without yeast phagocytised from each larva. Trials were performed at least three times on different days.

### 2.5. Antifungal Treatments with Echinocandins

Groups of 20 larvae of *G. mellonella* were inoculated with 1 × 10^6^ cells/larva. These infected larvae were treated with the echinocandins, AND (Pfizer SA, Madrid, Spain), CAS (Merk & Com Inc., Kenilworth, NJ, USA) and MCF (Astellas Pharma Inc., Tokyo, Japan) at concentrations of 4 and 8 μg/g larva. The stock solutions of the three echinocandins were dissolved in dimethyl sulfoxide (DMSO) according to the manufacturer’s recommendations, and then, the doses of antifungal drugs for treatments were prepared with PBS-ampicillin. The treatments were administrated with the pathogen inocula in a final volume of 10 μL per larva, 20 larvae were included in each condition. Moreover, three control groups with uninfected larvae were included, a group of untouched larvae, a group of larvae injected with PBS-ampicillin (sham group), and a third group of larvae injected with each antifungal drug to evaluate their possible toxicity. The larvae were incubated at 37 °C in dark for 120 h, and survival was monitored every 24 h by visual inspection of melanisation and the absence of movement. Each trial was performed at least three times on different days. A total of 780 larvae were used to assess the effect of the different treatments against each of the six *Candida* strains and 100 larvae were used as control in each trial.

### 2.6. Statistics

The results obtained of haemocytes production and phagocytic activity were analysed using one-way ANOVA with the statistical program SPSS v24.0 (IBM, Chicago, IL, USA). Survival analysis curves were prepared with the Kaplan-Meier method using GraphPad Prism 5 software (GraphPad Software, San Diego, CA, USA). Differences in *G. mellonella* survival infected with the *Candida* strains and exposed to the different antifungal treatments were analysed by the log-rank test using SPSS v24.0. The value of *p* < 0.05 was considered as statistically significant.

## 3. Results

### 3.1. Virulence of Candida in the G. mellonella Model

*C. glabrata*, *C. nivariensis* and *C. bracarensis* caused invasive candidiasis in the *G. mellonella* model. Significant differences were observed in the survival of infected and uninfected larvae used as controls (*p* ≤ 0.003). Survivals of untouched larvae and those PBS-ampicillin injected larvae were 87% ± 1.45% and 88.5% ± 1.5%, respectively, without significant differences among them (*p* = 0.663).

**Figure 1:**
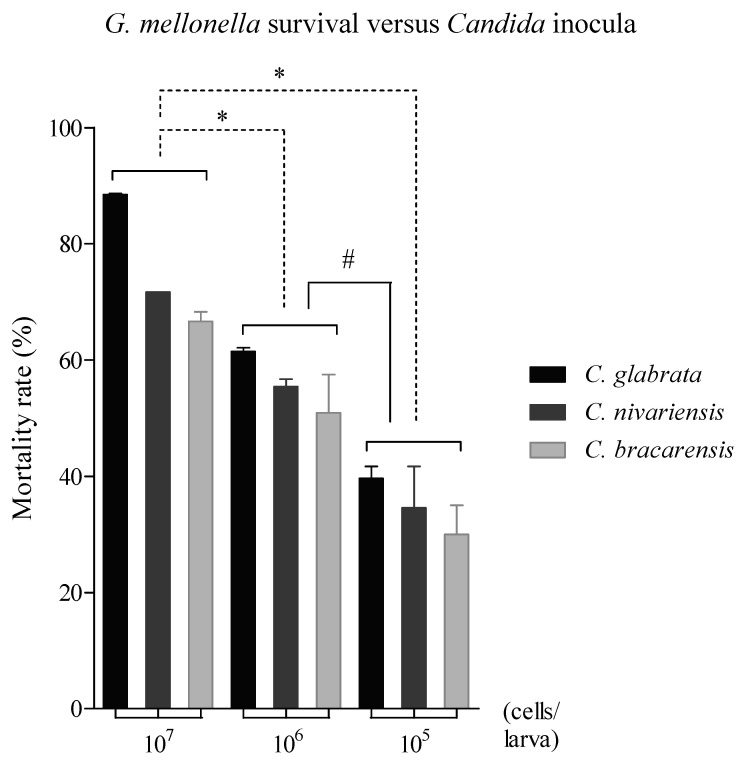
Mortality rate at 120 h post-infection of *G. mellonella* larvae infected with 1 × 10^7^, 1 × 10^6^ and 1 × 10^5^ cells/larva of *C. glabrata*, *C. nivariensis* and *C. bracarensis* strains. Statistically significant differences compared to larvae infected with the inoculum 1 × 10^7^ cells/larva (*) and, between inocula 1 × 10^5^ and 1 × 10^6^ cells/larva (#).

Three inocula were assayed to evaluate the virulence of the strains of *C. glabrata*, *C. nivariensis* and *C. bracarensis* observing that larval mortality was inoculum-dependent. Mortality rate was directly proportional to the injected inoculum: 34.7% ± 7.1%, 55.9% ± 6.4% and 76.8% ± 10.3% from lowest to highest inocula, respectively. Moreover, there were significant differences among the three species (*p* ≤ 0.001) (Figure 1). The virulence of these species in the *G. mellonella* model was categorised as *C. glabrata* > *C. nivariensis* > *C. bracarensis*, in all the cases regardless of the inoculum applied. However, there were mortality rate differences among the injected inocula with the strains of each species.

**Figure 2:**
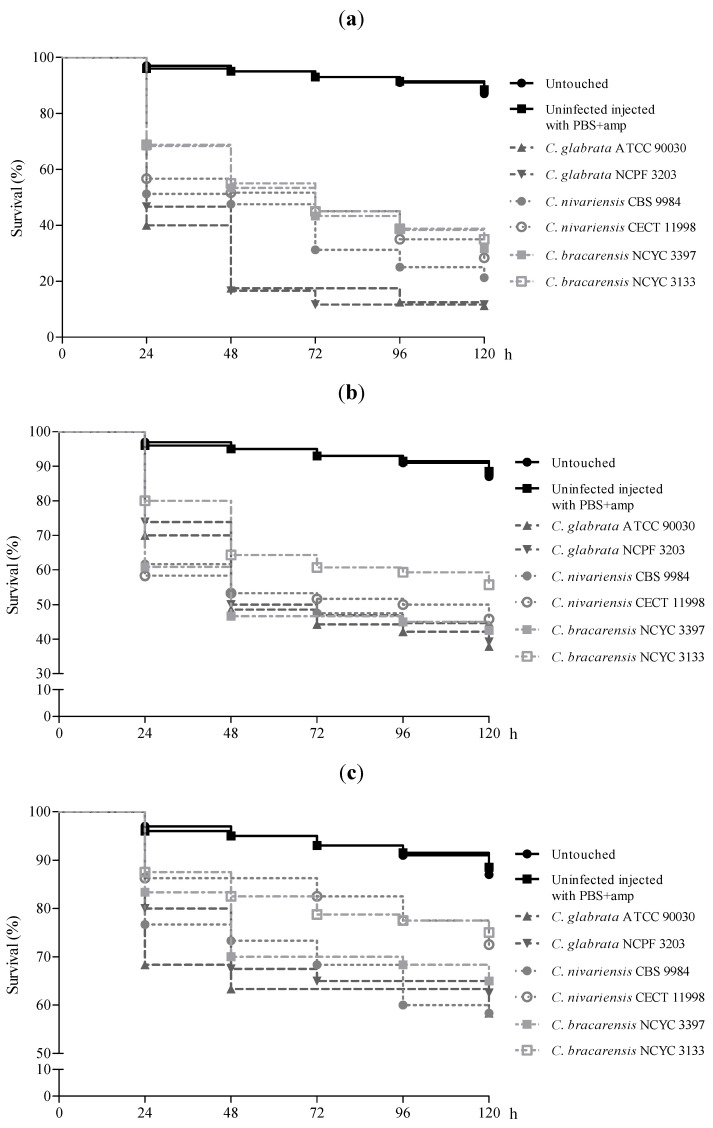
Survival curves of *G. mellonella* infected with 1 × 10^7^ cells/larva (**a**), 1 × 10^6^ cells/larva (**b**), and 1 × 10^5^ cells/larva (**c**) of *C. glabrata*, *C. nivariensis* and *C. bracarensis* strains.

The infection caused by 1 × 10^7^ cells/larva showed that *C. glabrata* was significantly more virulent than *C. nivariensis* (*p* ≤ 0.024) and *C. bracarensis* (*p* ≤ 0.001), showing differences in their killing kinetics. At 48 h of infection, larvae inoculated with *C. glabrata* achieved mortality rates above 80%, while the mortality rate was 50–60% for those inoculated with *C. nivariensis* and *C. bracarensis*. However, during the next 72 h of infection, *C. glabrata* killed 8% more larvae, and *C. nivariensis* and *C. bracarensis* killed more than 20% (Figure 2a). There were also significant differences between the *C. nivariensis* CBS 9984 strain and the *C. bracarensis* NCYC 3133 strain, being the last one the least virulent of tested strains (*p* = 0.048).

Strikingly, there were no significant differences in virulence among the three species when larvae were inoculated with 1 × 10^6^ and 1 × 10^5^ cells/larva, except for the *C. bracarensis* NCYC 3133 strain that achieved the highest survival rate (55.7% and 75%, respectively) at 120 h post-infection. Survival of *G. mellonella* inoculated with 1 × 10^6^ cells/larva was lower than 55% at 48 h post-infection except with the *C. bracarensis* NCYC 3133 strain, which it took more than 120 h to reach a mortality rate of 44% (Figure 2b). This latter and the *C. nivariensis* CECT 11998 strains did not kill more than 28% of the larvae after 120 h with the inoculum of 1 × 10^5^ cells/larva, and the remaining *Candida* strains failed to kill more than 42% of the larvae (Figure 2c).

No differences in the larvae survival were found within the two *C. glabrata* strains or within both *C. nivariensis* strains, regardless of the inoculum used. However, the survival for larvae infected with 1 × 10^6^ cells of *C. bracarensis* strain NCYC 3397 was significantly lower than that of larvae infected with the *C. bracarensis* NCYC 3133 strain (*p* = 0.013).

### 3.2. Haemocyte Production during Candidiasis

**Figure 3:**
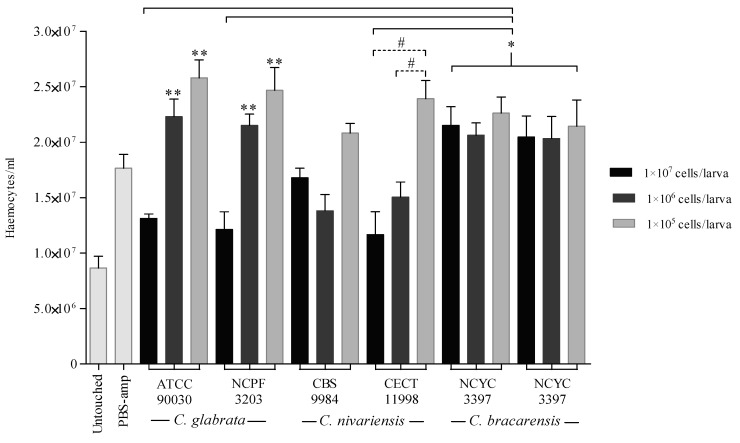
Haemocytes production from *G. mellonella* larvae infected with 1 × 10^7^, 1 × 10^6^ and 1 × 10^5^ cells/larva of *C. glabrata*, *C. nivariensis* and *C. bracarensis*. Statistically significant differences compared to larvae infected with *C. bracarensis* (*), 1 × 10^7^ cells/larva of *C. glabrata* (**), and 1 × 10^7^ and 1 × 10^6^ cells/larva of *C. nivariensis* strain CECT 1198 (#).

*G. mellonella* larvae were injected with 1 × 10^5^, 1 × 10^6^ and 1 × 10^7^ cells/larva and haemocytes density was calculated at 3 h post-infection (Figure 3). The injection of PBS-ampicillin and the two lowest inocula of all species, except 1 × 10^6^ cells/larva of both *C. nivariensis* strains (*p* ≤ 0.492), induced a significant increase (*p* ≤ 0.005) in the number of haemocytes compared with the control group of untouched larvae. It is noteworthy that the density of haemocytes from larvae infected with the highest inoculum (1 × 10^7^ cells/larva) of both strains of *C. glabrata*, and *C. nivariensis* strain CECT 11998 was significantly lower in comparison to that from larvae infected with both *C. bracarensis* strains (*p* ≤ 0.041).

Larvae infected with *C. bracarensis* showed the highest haemocyte density to overcome the infection caused with any of the three inocula tested, without significant differences among them. Neither was difference between the haemocyte densities of the larvae infected with the three inocula of the *C. nivariensis* CBS 9984 strain. However, larvae infected with 1 × 10^5^ and 1 × 10^6^ cells of *C. glabrata* showed a higher haemocyte number than those infected with 1 × 10^7^ yeasts (*p* ≤ 0.004).

### 3.3. Phagocytic Activity of G. mellonella Haemocytes during Candidiasis

Phagocytosis was evaluated with larvae infected with 1 × 10^6^ *Candida* cells/larva, which was considered, according to the results of haemocyte density determination, the most suitable inoculum because of the adequate number of haemocytes and yeasts.

**Figure 4:**
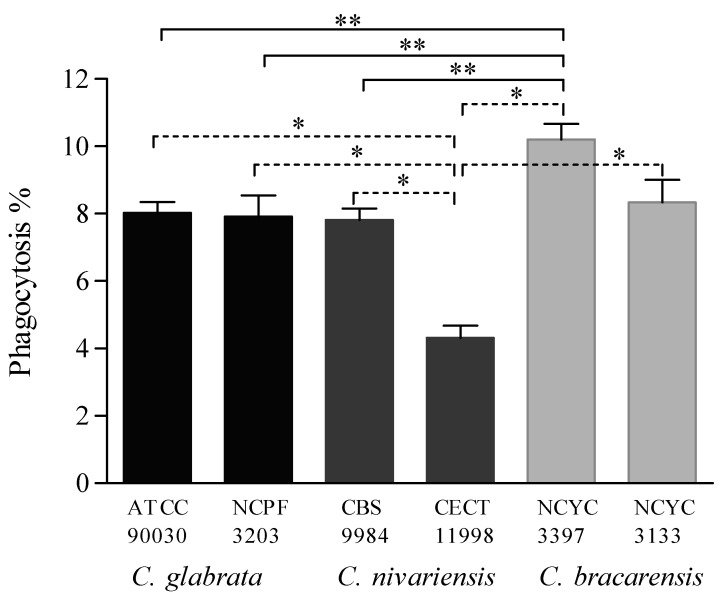
Phagocytic activity of *G. mellonella* at two hours post-infection with 1 × 10^6^ cells/larva of *C. glabrata*, *C. nivariensis* and *C. bracarensis*. Statistically significant differences compared to larvae infected with *C. nivariensis* CECT 11998 (*) and *C. bracarensis* NCYC 3397 (**).

The three *Candida* species were phagocytosed after 2 h of the infection (Figure 4). *C. bracarensis* was more effectively phagocytosed than *C. glabrata* and *C. nivariensis*. The percentage of haemocytes that phagocytosed *C. bracarensis* strain NCYC 3397 was the highest observed (11.26% ± 0.91%) and it was significantly different to the other two species (*p* ≤ 0.041). In contrast, cells of *C. nivariensis* strain CECT 11998 were the least phagocytosed (5.31% ± 0.71%, *p* ≤ 0.001).

### 3.4. Efficacy of Echinocandins Treatment of Invasive Candidiasis in G. mellonella

In a previous study we reported the in vitro antifungal activity of echinocandins against these six strains of *Candida*. All strains were susceptible to the three echinocandins tested. MIC values for AND of 0.06 µg/mL and 0.03 µg/mL for MCF were obtained against all strains. MIC values for CAS of 0.5 µg/mL were observed against the *C. glabrata* ATCC 90030 strain and 0.25 µg/mL against the other five *Candida* strains [15].

Treatment with three different echinocandins was analysed in vivo after the injection of 1 × 10^6^ cells/larva in *G. mellonella.* This inoculum was selected as the most appropriate because the larvae survival was similar during the infection by all six strains, so the effect of the antifungal treatment could be more accurately assessed. The antifungal drugs were not toxic for *G. mellonella*; survivals of larvae injected with antifungal drugs were 86% ± 3.4%, without significant differences among the other two controls (*p* ≥ 0.29).

**Figure 5:**
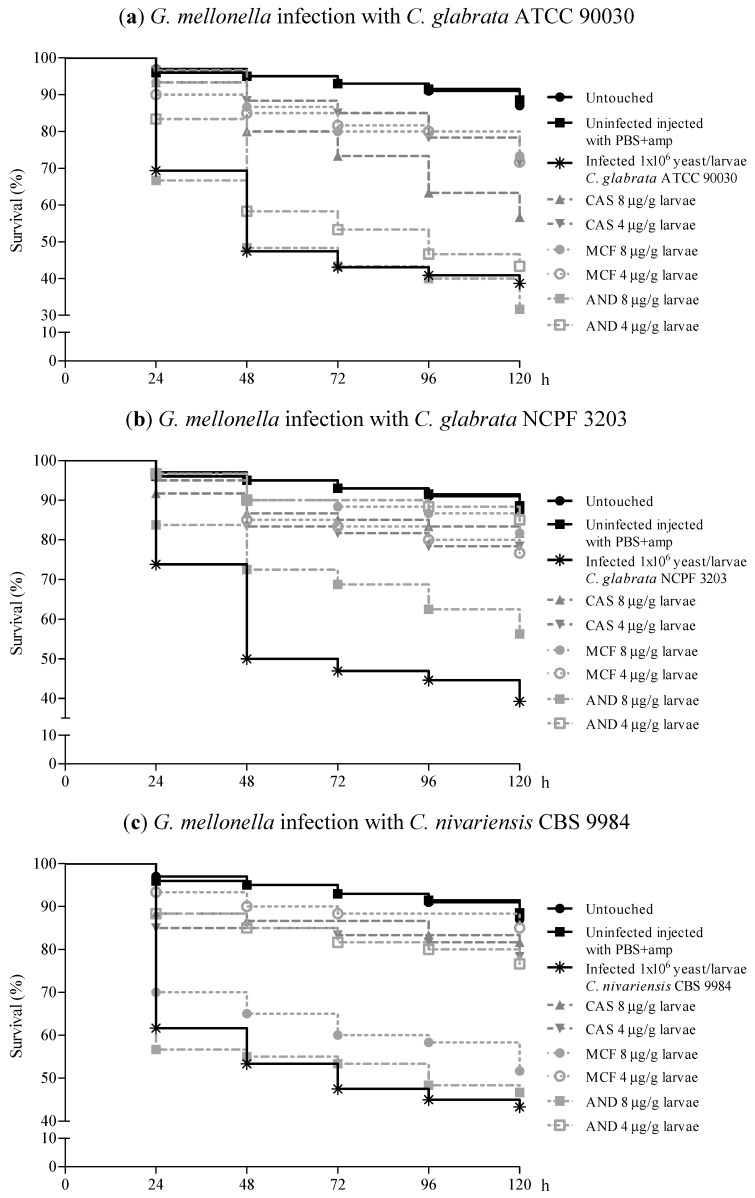

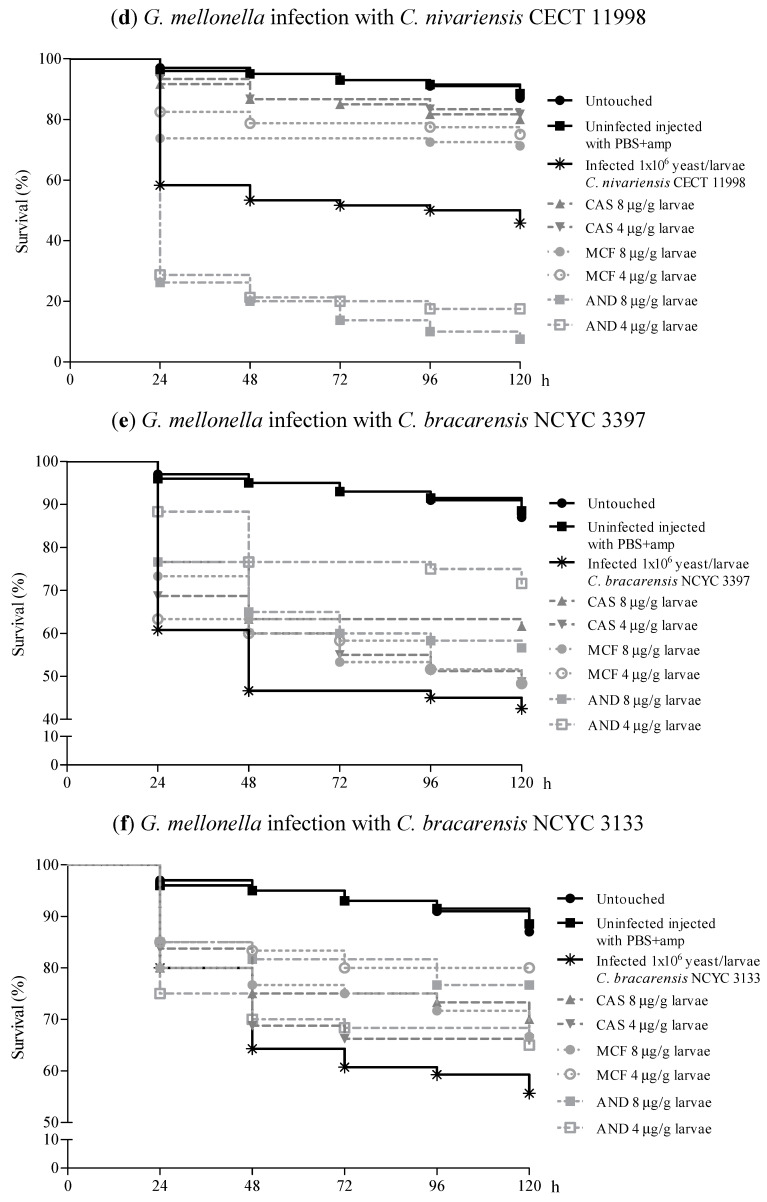
Activity of echinocandins in the treatment of *G. mellonella* infection by *C. glabrata* ATCC 90030 (**a**), *C. glabrata* NCPF 3203 (**b**), *C. nivariensis* CBS 9984 (**c**), *C. nivariensis* CECT 11998 (**d**), *C. bracarensis* NCYC 3397 (**e**), and *C. bracarensis* NCYC 3133 (**f**). Larvae were inoculated with 1 × 10^6^ cells/larva and treated with anidulafungin (AND), caspofungin (CAS) and micafungin (MCF) at concentrations of 4 and 8 µg/g larva.

Echinocandin treatment was very effective against *C. glabrata* candidiasis. After 120 h there was a reduction in larvae mortality of between 33% and 45.8% (Table 1). However, the efficacies of these echinocandins against *C. glabrata* infection were strain-dependent (Table 1 and Figure 5a,b).

Candidiasis caused by both *C. glabrata* strains responded to MCF at the two concentrations tested, achieving a significant increased larvae survival with respect to infected and untreated larvae (*p* = 0.000). Specifically, treatment with MCF (8 µg/larva) against ATCC 90030 strain infection increased larvae survival from 38.7 to 73.3%. During treatment against CBS 3203 strain candidiasis, survival increased from 39.2 to 81.7% compared to the survival of infected and untreated larvae.

CAS was also effective against *C. glabrata* candidiasis: the larvae survival was significantly higher than that of infected and untreated larvae (*p* ≤ 0.004). Treatment of NCPF 3203 strain infection with 4 µg/g larva of CAS reached a 39.1% increase in survival of larvae. This survival increase was higher (42.5%) using 8 μg/g larva of CAS. However, when candidiasis caused by ATCC 90030 was treated with 8 μg/g larva of CAS the survival of *G. mellonella* increased to 18%, while with 4 μg/g larva of CAS, it increased up to 33%.

*C. glabrata* NCPF 3203 infection only responded adequately to 4 µg/g larva of AND. An increase of larvae survival of 45.8% with respect to infected and untreated control group was achieved (*p* ≤ 0.011). The *G. mellonella* larvae infected with the ATCC 90030 strain showed no improvement with AND treatment. Although the mortality of larvae infected with this strain and treated with 8 μg/g larva of AND increased up to 7%, there were no differences in the survival when compared with infected and untreated control larvae group (Figure 5a).

*C. nivariensis* infection was successfully treated with MCF and CAS (Figure 5c,d). The survival rates of larvae infected with both strains of *C. nivariensis* and treated with CAS ranged from 61.6% to 65.8%, and there were significant differences compared to those infected and untreated larvae (*p* = 0.000). A statistically significant increase in larvae survival was also detected when *G. mellonella* infected with *C. nivariensis* was treated with MCF (*p* ≤ 0.001). Larvae survival increased up to 41.7% with 4 µg/g larva of MCF against the infection by the *C. nivariensis* CBS 9984 strain. Strikingly, this significant difference was not detected in larvae infected with the *C. nivariensis* CBS 9984 strain and treated with MCF at the highest concentration (8 µg/g larva) that only got a survival increase of 8.4%.

AND treatment of *C. nivariensis* infection was the least effective. Only 4 µg/g larva of AND significantly increased *G. mellonella* survival (33.4%) during infection with the *C. nivariensis* CBS 9984 strain (*p* = 0.000) (Figure 5c). The treatment of candidiasis by *C. nivariensis* strain CECT 11998 with AND even increased the larvae mortality (28.3% with 4 µg/g larva of AND, and 38.3% with 8 µg/g larva of AND) (Figure 5d).

*C. bracarensis* infection was the least susceptible to treatment with echinocandins (Figure 5e,f). It is noteworthy that AND was the most effective during *G. mellonella* infection with this *Candida* species. The concentration of 4 µg/g larva of AND presented the highest protective effect during larvae infection with the *C. bracarensis* NCYC 3397 strain: infected and untreated larvae survival was 42.5% and treatment with AND increased survival to 71.7%. The therapeutic agents MCF (4 µg/g larva) and AND (8 µg/g larva) reached also high effect against *C. bracarensis* NCYC 3133 infection: larvae survival rate significantly increased by 24.3% (*p* = 0.002) and 21% (*p* = 0.008), respectively. Although the rest of MCF treatments achieved mortality reductions between 5.8% and 11%, there were no differences with the infected and untreated control group. The antifungal agent CAS during *C. bracarensis* infection reduced the larvae mortality rate between 6.3% and 19.2%, and there were also no differences with untreated larvae, except in larvae infected with the *C. bracarensis* NCYC 3397 strain and treated with 8 µg/g larva of CAS (19.2% of survival increase) (*p* = 0.015).

## 4. Discussion

Alternative animal models, such as those in *C. elegans* and *G. mellonella*, have been explored as a useful option to study the pathogenesis and treatment of invasive candidiasis. The use of *G. mellonella* has acquired relevance as larva size makes possible to control the infection development and treatment more easily, since it allows the injection of microorganism suspensions and antimicrobial treatments at specific concentrations [19,25,26,29]. However, there are few studies about *C. glabrata*, *C. nivariensis* and *C. bracarensis* candidiasis using in vivo non-mammalian models [18,20,22]. These three species present similar phenotypic characteristics and are difficult to differentiate from each other without molecular methods based on PCR, sequencing or MALDI_TOF MS; they are taxonomically different, and their pathogenesis and antifungal susceptibility can also be very different. Although the incidence of candidiasis caused by *C. nivariensis* and *C. bracarensis* is low, several authors consider that the difficulty in achieving a correct identification may be causing a misjudgment of the real medical importance of these species [2,3,4]. In addition, the emergence of isolates resistant to antifungal drugs, such as amphotericin B, fluconazole, voriconazole and even echinocandins, makes it necessary to deepen the knowledge on *C. glabrata* and these two closely related species [15,30,31,32,33].

Therefore, in the present study the usefulness of *G. mellonella* model to assess the pathogenicity of *C. glabrata*, *C. nivariensis* and *C. bracarensis* was analysed. The six strains studied of these *Candida* species developed invasive candidiasis in larvae of *G. mellonella*: *C. glabrata* and *C. bracarensis* were the most and less virulent species, respectively, just as we had previously observed in a model of candidiasis in *C. elegans* in which *C. glabrata* killed the highest percentage of nematodes followed by *C. nivariensis* and *C. bracarensis* [20].

A notable difference between these two models, *C. elegans* and *G. mellonella*, is the yeast inoculum administered. In the case of *G. mellonella* model, *Candida* cells are injected into haemolymph, allowing a more precise control and better knowledge of the effect of yeast inocula [29]. In an attempt to detect the most appropriate inoculum, three different *Candida* concentrations were evaluated. The highest concentration (1 × 10^7^ cells/larva) showed significant virulence differences between the three *Candida* species. Ames et al. [18] also reported that the highest injected dose tested (7.5 × 10^6^ cells/larva) was the best for studying *C. glabrata* virulence in *G. mellonella*. Nevertheless, other *Candida* species such as *C. albicans*, *C. tropicalis*, *C. krusei* and *C. parapsilosis* complex required lower yeast inocula to cause candidiasis in this model, even detecting higher larvae mortality rates than those observed for *C. glabrata* [21,22,25]. Furthermore, and in agreement with previous studies, an increase in mortality was observed in *G. mellonella* as the injected fungal load was higher [18,19,26].

An additional highlight of the *G. mellonella* model of invasive candidiasis is the analysis of host-pathogen interactions. This lepidopteran uses different mechanisms to combat pathogens, such as a variable production of haemocytes and the phagocytic activity of haemocytes according to the virulence of pathogens [33]. Larvae of *G. mellonella* infected with *C. glabrata* and *C. nivariensis* produced fewer haemocytes than those infected with *C. bracarensis*, and the latter species was the most effectively phagocytized by haemocytes. This was consistent with the ability of these three species to develop infection in *G. mellonella*. Several authors have attributed the decrease of haemocytes in haemolymph to the formation of nodules at the sites of infection in order to contain the spread of pathogens [19,26,33]. The haemocyte production observed in other studies during the infection caused by *C. albicans*, *C. krusei*, *C. tropicalis*, *C. parapsilosis* or *C. orthopsilosis* was even lower, likely due to a higher virulence of these species [19,25,26]. *C. bracarensis* as well as other not so virulent species, such as *C. metapsilosis*, generated a low *G. mellonella* haemocyte response. Although the phagocytic rate detected in larvae infected with *C. parapsilosis* complex was higher than that detected with *C. glabrata*, a significant difference was observed in the less virulent species, *C. metapsilosis* and *C. bracarensis*, respectively, compared to other close-related species [25]. Nevertheless, it has been reported that the presence of *C. glabrata* enhances the activity of *G. mellonella* haemocytes enough to protect larvae against subsequent lethal fungal infections by *C. albicans*, *C. tropicalis* and *Cryptococcus neoformans* [21]. The increased expression of redox related proteins, the presence of multidrug transporter such as CgTpo4 or the expression of the transcription factor CgTog1 to survive upon phagocytosis are determinant mechanisms of virulence recently described in *C. glabrata*; and this species requires them to survive host defence, quickly develop resistance to drugs and kill *G. mellonella* [23,24,34,35]. On this issue, both species closely related to *C. glabrata*, *C. nivariensis* and *C. bracarensis*, are very poorly studied, therefore, their research using the great potential of the *G. mellonella* model of candidiasis is promising and encouraging.

In vitro antifungal susceptibility has been widely studied in *C. glabrata*, *C. nivariensis,* and *C. bracarensis*, indicating a reduced susceptibility or even resistance to azoles and amphotericin B [30,31]. Specifically, the six strains of these three closely related species, used in the current study, were in vitro susceptible to amphotericin B, azoles (posaconazole and voriconazole) and all three echinocandins [20].

Echinocandins are mainly indicated for the treatment of candidiasis caused by *C. glabrata* [11,36,37]. However, an increase in echinocandin resistance has been described, mainly due to acquired *FKS* mutations associated to previous exposure to these drugs. Although the incidence of these mutations is still low, it could be useful to know the local resistance patterns to establish adequate empirical treatment strategies [7,8,17]. Echinocandins treatment was effective against the infection caused by these three *Candida* species in *G. mellonella* host model. CAS and MCF showed in vivo efficacy during *C. glabrata* and *C. nivariensis* infections while AND during *C. bracarensis* candidiasis. These findings are strongly consistent with those previously observed in the *C. elegans* model of candidiasis [20]. Treatment with CAS protected the larvae during *C. glabrata* infection, maintaining survival above 72% after 120 h infection. Ames et al. [18] also detected up to 80% survival rate in *C. glabrata* infection treated with 4 µg/g larva of CAS. This effectiveness of CAS has also been reported in *C. glabrata* murine models of invasive candidiasis [38,39,40]. However, Healy et al. [41] reported that administration of high doses of CAS (20 mg/kg) to murine for 5–9 days selected *C. glabrata* strains with *FKS* mutations resistant to echinocandins. Although in time-kill studies echinocandins showed a lower effect against *C. nivariensis* [42], we observed that CAS achieved a reduction in larval mortality during *C. nivariensis* infection. Lopez-Soria et al. [43] observed that CAS was very effective in resolving a catheter-associated fungemia caused by *C. nivariensis*. Moreover, *C. nivariensis* and *C. bracarensis* have been susceptible to CAS in vitro [15,17,31].

The effectiveness of treatment with MCF of candidiasis caused by *C. glabrata* in murine was also reported [44,45]. However, in these studies a higher MCF concentration than those used of CAS was required to achieve the same effect. In the present study, MCF was also effective against *C. nivariensis* infection confirming the in vitro susceptibility of this species to the MCF [15,38,46].

Treatment with AND was the least effective against infection caused by *C. glabrata* and *C. nivariensis* in *G. mellonella*. Strikingly, larvae infected with *C. glabrata* ATCC 90030 and *C. nivariensis* CECT 11998 strains and treated with AND showed an increase in mortality up to six times higher than that of infected and untreated larvae. This lower effect of AND treatment has also been reported in a murine model of *C. glabrata* infection [38]. However, infected larvae treated with AND showed a reduction in mortality rates higher than with CAS and MCF in *C. bracarensis* infection in *G. mellonella*. This is consistent with observations reported in other *C. bracarensis* candidiasis models and in vitro susceptibility [15,20,31].

In conclusion, our findings demonstrate that *G. mellonella* is a suitable model to analyse the virulence and host-pathogen interactions caused by emerging species of *Candida* and to assess the efficacy of echinocandins as therapy. This study contributes to encourage future research to extend this model in the studying of candidiasis by *C. albicans* and other emerging *Candida* species.

## Figures and Tables

**Table 1:** Survival of *G. mellonella* larvae infected with 1 × 10^6^ cells/larva of *C. glabrata*, *C. nivariensis* and *C. bracarensis* with and without treatment with echinocandins.

Strain	Origin	Collection Reference	Survival Percentages of *G. mellonella* at 120 h	Most Effective Antifungal Treatments (Survival Rate Increase of *G. mellonella* at 120 h)
***Candida glabrata***				
ATCC 90030	Blood	American Type Culture Collection	38.7%	Micafungin, 8 µg/larva (34.6%) Micafungin, 4 µg/larva (33%) Caspofungin, 4 µg/larva (33%)
NCPF 3203	Blood	National Collection of Pathogenic Fungi	39.2%	Anidulafungin, 4 µg/larva (45.8%) Micafungin, 8 µg/larva (42.5%) Caspofungin, 8 µg/larva (42.5%)
***Candida nivariensis***				
CBS 9984	Bronchoalveolar lavage	Westerdijk Fungal Biodiversity Institute	43.3%	Micafungin, 4 µg/larva (41.7%) Caspofungin, 8 µg/larva (38.4%) Caspofungin, 4 µg/larva (35%)
CECT 11998	Blood	Spanish Type Culture	45.8%	Caspofungin, 4 µg/larva (35.9%) Caspofungin, 8 µg/larva (34.2%) Micafungin, 4 µg/larva (29.2%)
***Candida bracarensis***				
NCYC 3397	Blood	National Collection of Yeast Cultures	42.5%	Anidulafungin, 4 µg/larva (29.2%) Caspofungin, 8 µg/larva (19.2%) Anidulafungin, 8 µg/larva (14.2%)
NCYC 3133	Catheter	National Collection of Yeast Cultures	55.7%	Micafungin, 4 µg/larva (24.3%) Anidulafungin, 8 µg/larva (21%) Caspofungin, 8 µg/larva (14.6%)
